# Named entity recognition for Chinese based on global pointer and adversarial training

**DOI:** 10.1038/s41598-023-30355-y

**Published:** 2023-02-24

**Authors:** Hongjun Li, Mingzhe Cheng, Zelin Yang, Liqun Yang, Yansong Chua

**Affiliations:** 1 Key Laboratory of Deep-time Geography and Environment Reconstruction and Applications, MNR & College of Computer Science and Cyber Security, Chengdu University of Technology, Chengdu, 610059 China; 2 College of Computer Science and Cyber Security, Chengdu University of Technology, Chengdu, 610059 China; 3 China Nanhu Academy of Electronics and Information Technology CNAEIT, Jiaxing, 314001 China

**Keywords:** Computer science, Information technology

## Abstract

Named entity recognition aims to identify entities from unstructured text and is an important subtask for natural language processing and building knowledge graphs. Most of the existing entity recognition methods use conditional random fields as label decoders or use pointer networks for entity recognition. However, when the number of tags is large, the computational cost of method based on conditional random fields is high and the problem of nested entities cannot be solved. The pointer network uses two modules to identify the first and the last of the entities separately, and a single module can only focus on the information of the first or the last of the entities, but cannot pay attention to the global information of the entities. In addition, the neural network model has the problem of local instability. To solve mentioned problems, a named entity recognition model based on global pointer and adversarial training is proposed. To obtain global entity information, global pointer is used to decode entity information, and rotary relative position information is considered in the model designing to improve the model’s perception of position; to solve the model’s local instability problem, adversarial training is used to improve the robustness and generalization of the model. The experimental results show that the F1 score of the model are improved on several public datasets of OntoNotes5, MSRA, Resume, and Weibo compared with the existing mainstream models.

## Introduction

Named entity recognition (NER) is one of the significant task of Natural language processing (NLP) and knowledge graph (KG) construction, whose aim is recognizing the entity from unstructured text.

In the medical field, medical dialogue contains information about the patient’s symptoms and the doctor’s diagnosis. The entities in the medical dialogue are medical proper names such as symptoms, drug names, drug categories, medical tests and operations. For example, in the following doctor-patient dialogues, “Does the child have fever and vomiting?” and “Can you treat the child with nebulizer inhalation?”, where “fever” and “vomiting” are symptoms and“nebulizer inhalation”is the operation. Named entity recognition is the process to identify the specific classes of words.

The main solution for this task is based rule and dictionary in the early age, SRA^1^, FASTUS^2^, LTG^3^. These methods intend to induct rules and create a dictionary based on a specific grammar or task for NER. However, because of the limitation of the rule and dictionary, the model transposing between tasks is tough.

With the developing of machine learning, the method based on hidden markov model (HMM)^4^, support vector machine (SVM) and conditional random fields (CRF) are widely utilized in this field. The performance of most of these methods depend on the feature template and training data. Moreover, complex manual work and data starving limit the development of these methods.

With the rise of the third artificial intelligence tide, the neural network based methods dominate this field in a short time^5–9^. Li et al.^5^ provide a novel method for NER with Long Short-Term Memory (LSTM) and Convolutional Neural Network (CNN). Rabiner^4^ involves attention mechanism and atrous convolution.

For Named Entity Recognition, deep learning methods use neural network models such as^10^ which uses Long Short-Term Memory Networks (LSTM), and which applies attention mechanisms and hold-up convolution. In deep learning, named entity recognition tasks are typically labeled in two ways sequential labeling^5–8^ and Pointer labeling^9^. In the former, each token in a sentence is labeled with a tag. The advantage of this approach is that it is faster when there are fewer entity categories but more costly when the number of entity categories is large. However, the drawback is that a token is labeled with a single tag, while in reality, a token may belong to multiple entities or different types of entities, so this approach does not solve the nested entity issue. The later, pointer labeling, focuses only on the head and tail position of an entity, which has the advantage of solving the nested entity problem. The disadvantage is that each pointer network uses two modules to identify the head and tail of an entity separately, and individual modules can only focus on the head or tail of an entity, not the overall information of an entity.

It contains three modules in this model, encode module, decode module, adversarial training module. In the encode module, we use Chinese-BERT as the sentence encoder, and use the pinyin information to do the knowledge enhancement for the embedding layer of the model, and also use the relative position encoding in the attention mechanism to inject position information into the model to enhance the positional relationship between entities. In the decode module, we use the global pointer as the label decoder. In the adversarial training module, we use adversarial training to add perturbations to generate adversarial samples to improve the robustness and generalization of the model.

The main contributions of this article are as follows:

1. A novel end-to-end model is proposed to decode entity information using global pointer network, which can focus on global information better.
2. Our work verifies two strategies can improve the performance of the model related to Chinese NER, rotary relative position coding and adversarial training.
3. Through experiments on multiple open-source datasets, we demonstrate that the AT-CBGP model achieves better results on F1 score than existing mainstream ones.

## Related works

Most existing naming entity recognition methods use sequence tagging. Zhai et al.^11^ used BILSTM to extract text messages and input text messages in both directions, which enabled the model to take context into account and finally decode the hidden layer by employing CRF. To compensate for the inability of BiLSTM-CRF model to capture the dependency of long distances between the two entities. Zeng et al.^12^ proposed a BiLSTM-CRF model combining self-attention. Zhang et al.^8^ proposed the BERT-BiLSTM-IDCNN-CRF model for initial mathematical text entity recognition, which uses BERT, then combines the output of iterative expansive convolution network with BiLSTM, and put it into the CRF layer.

In addition, the lexical enhancement method has also been widely studied^13^. Zhang et al. proposed the Lattice LSTM^14^ model, which injects lexical information into LSTM and combines word-level and word-level information, but Lattice LSTM is not parallel and can’t effectively deal with lexical conflicts. Both problems are addressed by Gui et al. proposed the LR-CNN^15^ model, in which sentences are coded using CNN rather than RNN, and words of different lengths are incorporated into the model at different levels. Sui et al. proposed the Collaborative Graph Network (CGN^16^) model, which combines dictionary and character information in Encoding Layer, and proposes a graph layer and three graph structures to encode using graph attention networks, also to solve the problem that Lattice LSTM does not contain self-matching word information and the closest word information. However, there are still differences in the results between the graph structure and sequence structure because of the differences between the graph structure and sequence structure.

In addition to lexical information, studies have shown that glyph information, pinyin information and relative position information between glyph and glyph can improve model performance. Sun et al. proposed the Chinese BERT^17^ model, which can enhance the modeling ability of Chinese corpus by incorporating glyph and pinyin embedded information into the model. BERT first proposed the use of absolute location code to increase word embedding information, but the location code cannot represent the relative position information between words. In response, Shaw et al.^18^ propose relative location codes to better represent location information. Inspired by relative location information, Li et al.^19^ designed a new relative location code for FLAT to represent the position relationship between words. It transforms lattice structures into flat structures, adds words matched to sentences to the end of sentences, and uses Transformer^20^ to compute self-attention fusion word information into models. This method performs well in multiple public datasets. Further, Sue et al proposed the RoFormer^21^ model, which uses rotary relative position embedding (ROPE) to achieve relative position embedding using absolute position embedding to better represent the position relationship between token in a sequence.

Existing entity recognition methods and models mentioned above are sequenced and use conditional random fields decoder in decoding parts. The disadvantage of sequential tagging is that nesting entities cannot be identified well. Wei et al.^9^ proposed the CASREL model, which uses pointer annotation to extract a triad of objects, first subobjects and then objects in specified relationships, to better address nested entities.

In addition, studies^22^ have shown that fitting is easy to occur in model training due to the small size of data sets in specialized fields, and that most existing studies mitigate fitting using the Dropout method. Dropout is essentially a disturbance method^23^. Miao et al.^22^ propose to improve the robustness of the Entity Identification Model by combining Dropout and adversarial training to enhance the diversity of disturbances. Confrontation training as a regularization method was first used in the field of imagery^24^. Scholars Takeru^25^ and Giannis^26^ extended adversarial training to text sorting and relationship extraction tasks, respectively. Qiu et al.^27^ generates adversarial examples for spatiotemporal data and successfully attacks the deep learning model for the Travel Time Estimation (TTE) task and generates adversarial examples for GPS data using a computer vision algorithm to attack the trajectory model detection models, and small perturbations successfully fool the neural network with high confidence^28^.

Sequence tagging method has the following disadvantages:

1. The conditions of use of the method depend on conditional random fields as a label decoder, and when the number of labels is too large, the calculation cost is higher; hard to solve.
2. The pointer tagging method uses multiple pointer networks for multiple entity relational categories. Each pointer network uses two modules to identify an entity’s head and tail, and single module training assessments can only focus on the head or tail of an entity and not the overall information of an entity.
3. The neural network model has local instability, and the robustness and generalization of the model are poor.

Therefore, we propose a named entity recognition model AT-CBGP (Adversarial Training with Chinese BERT-base + GP) based on global pointer and adversarial training. The idea of this method is:

1. To treat entity extraction problem as character to link problem, we use global pointer network to extract global entity, obtain entity category information more comprehensively.
2. We use the rotary relative position code to inject position information into the model, so that the model can better distinguish the distance information between word and word, entity and entity.
3. We use adversarial training to add perturbations to the word embedding of the model to generate adversarial samples, and enhance the robustness and generalization of the model.

The results show that the model improve F1 score on the Onto Note 5, MSRA, Resume, Weibo open dataset compared to existing mainstream models.

**Figure 1:**
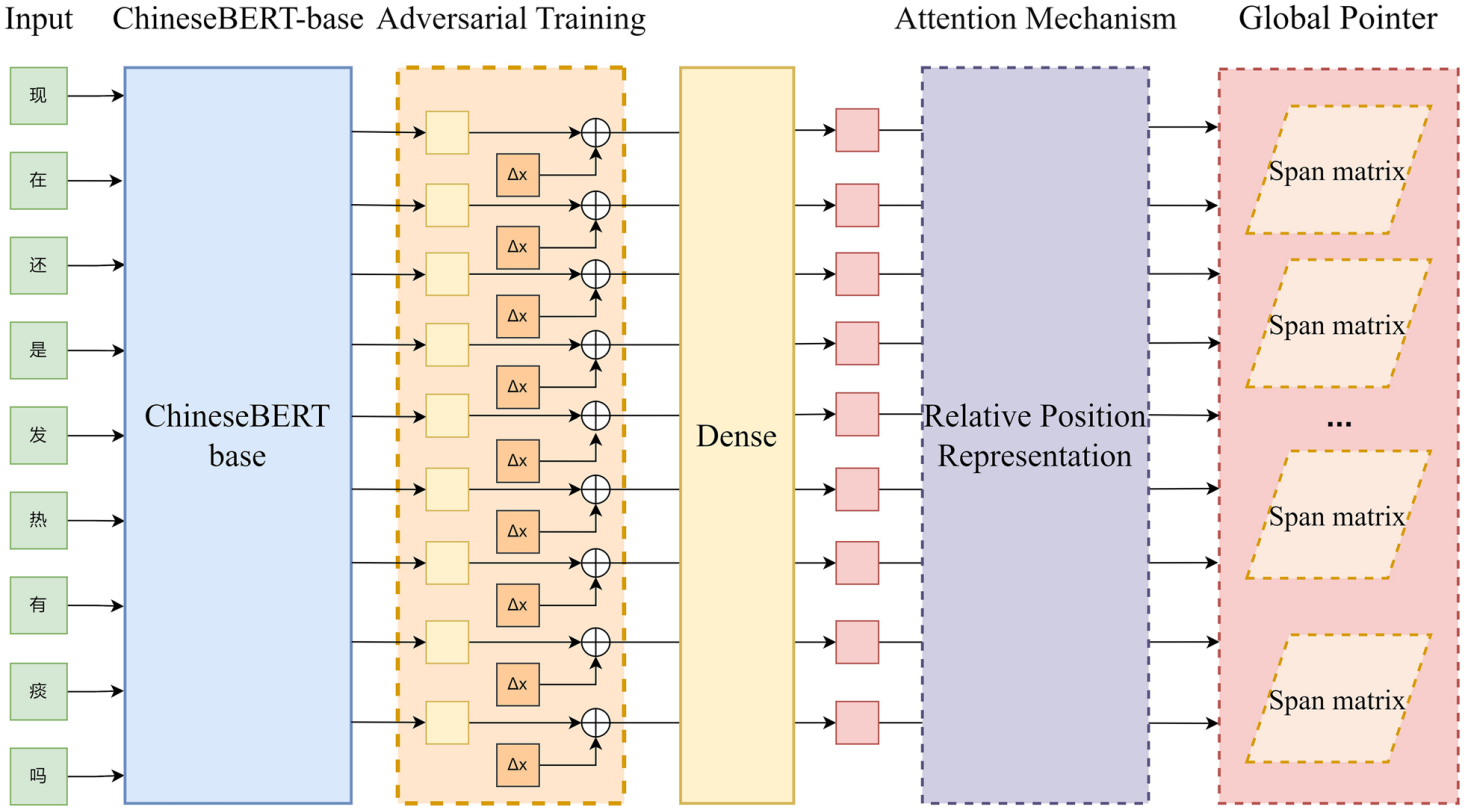
The Architecture of AT-CBGP: The AT-CBGP is a Bert-based model. The output of the model is the NER results in span matrix form for given input string.

## AT-CBGP

The overall architecture of the named entity recognition model (AT-CBGP) based on global pointer and adversarial training presented in this paper is shown in Fig. 1. Main technique used in this paper are Chinese BERT-base, adversarial training, attention mechanisms, and Global Pointer.

Among them, the Chinese BERT-base module encodes the input sequence $S = {c_1,c_2,...,c_n}$ with length *n* to get vector $X = {x_1,x_2,...,x_n}$, the adversarial training module adds perturbation $\Delta x$ to vector *X* to get the adversarial sample $X_{adv}$, then encodes the original vector *X* and the adversarial sample $X_{adv}$ through the linear layer module to get vector $H = {h_1,h_2,...,h_n}$, then enters vector *H* into the attention mechanism module to get vector *Q* and *K*, and vector *Q* and *K* do the internal product to get the attention score. In the attention module, relative position information is added to vectors *Q* and *K* using relative position coding. Finally, the output of the attention module is decoded into several Span matrices through the global pointer module.

### Chinese-BERT

Wu et al.^29^ suggests that Chinese characters evolve from hieroglyphics, and that their structure often reflects underlying text information. Character features enable models to better capture semantic information about Chinese characters. In addition, Sun et al.^17^ shows that the glyph and pinyin information of Chinese characters can enrich the word embedding of Chinese characters to represent information. Therefore, this paper uses chinese bert to implement words and pinyin embedding. Chinese BERT-base contains 12 layers of Transformer, each containing 12-heads of self-attention, and 768 hidden layer units. Suppose the input length is *n* for the sequence $S={c_1,c_2,...,c_n}$, $c_t$ for the *t* word in the sentence.

### Attention mechanism

Position encoding in the attention mechanism can be divided into absolute position encoding and relative position encoding. Although absolute position encoding can add position information to the word vector, this position information is associated with a fixed position and cannot represent the contextual information of the fixed position. Therefore, in order to represent the contextual information, Shaw et al.^30^ proposed relative position coding, which does not depend on the fixed position, but only on the relative position. In this paper, rotary relative position embedding (ROPE^21^) is used to integrate relative position information into the model to make the model more sensitive to the relative position between entities and can better identify them.

Rotary relative position encoding uses absolute position encoding to obtain the effect of relative position encoding by the nature of complex number operations. Suppose there are *C* entity classes, and a particular entity type is denoted as $\alpha$. $Q=\left[ \textbf{q}_{1, \alpha }, \textbf{q}_{2, \alpha }, \ldots , \textbf{q}_{n, \alpha }\right]$ and $K=\left[ \textbf{k}_{1, \alpha }, \textbf{k}_{2, \alpha }, \ldots , \textbf{k}_{n, \alpha }\right]$ in the attention mechanism are used to identify class $\alpha$ entities, and the inner product of $\textbf{q}_{i,\alpha }$ and $\textbf{k}_{j,\alpha }$ is done to obtain the attention score $score_{\alpha }(i,j)$, which represents the score of the input sequence *S*, where the subsequence consisting of the $i{\text{th}}$ element to the $j{\text {th}}$ element is the class $\alpha$ entity. Rotary relative position encoding introduces specific matrices $\textbf{R}_i$ and $\textbf{R}_j$. By constructing specific matrices $\textbf{R}_i$ and $\textbf{R}_j$ with respect to positions *i* and *j*, the score of a specific class of entities is obtained by multiplying matrices $\textbf{R}_i$ and $\textbf{R}_j$ with vectors $\textbf{q}_{i,\alpha }$ and $\textbf{k}_{j,\alpha }$ located at *i* and *j* respectively. The attention score depends only on the relative positions (*j*, *i*), i.e. the attention score contains relative position information.

1 $$\begin{aligned} score_{\alpha }(i,j) = (\textbf{R}_i\textbf{q}_{i,\alpha })^{\top }(\textbf{R}_j\textbf{k}_{j,\alpha }) = \textbf{q}_{i,\alpha }^{\top } \textbf{R}_i^{\top }\textbf{R}_j\textbf{k}_{j,\alpha } = \textbf{q}_{i,\alpha }^{\top } \textbf{R}_{j-i}\textbf{k}_{j,\alpha }. \end{aligned}$$

**Figure 2:**
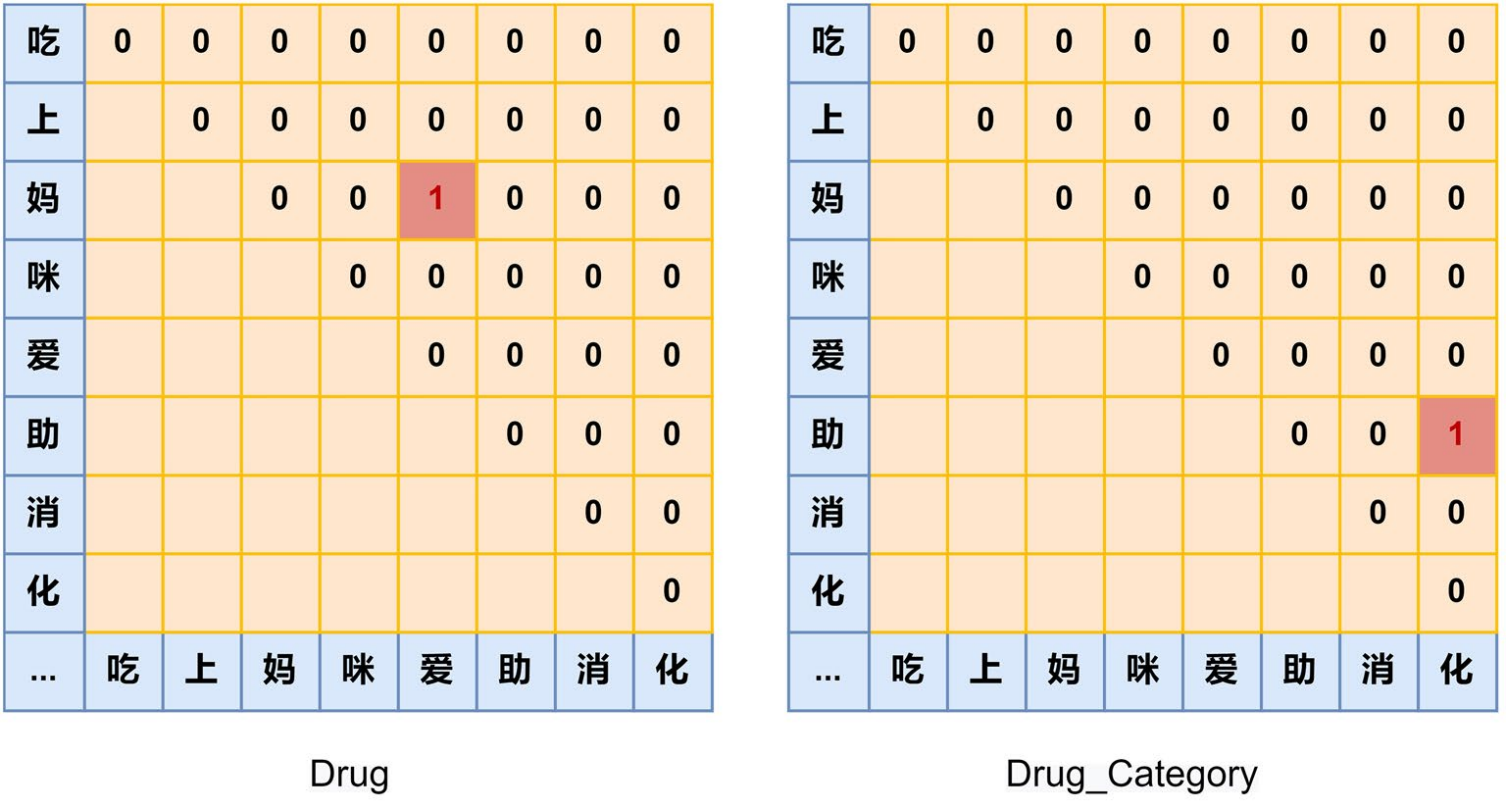
A demo of global pointer.

### Global pointer

This paper uses global pointer method^31,32^ to decode the output of attention module. The global pointer can solve the problem of nested entities compared to the condition of airfields. Compared with the general pointer network, the global pointer contains global information of an entity, which avoid incosistency in training and prediction.

The basic idea of the global pointer module is to identify entities as basic units. For sentences of *seq* length, it builds *C* span matrix the size of $seq \times seq$, which corresponds to a matrix for each type of entity in the sentence, *C* being the entity category. The row index of the mark is the head positiion of an entity and the column index of the mark is the tail position. Figure 2 shows a matrix corresponding to two types of entities in the sentence.

The global pointer module sees entity recognition as a multi-label classification problem. However, there are usually only a few partial category entities in a sentence, which causes category imbalance. To solve this problem, we use the uniform loss function form proposed in the literature Circle Loss^33^:

2 $$\begin{aligned} L_{u n i}=\log \left( 1+\sum _{i \in \Omega _{{\text {neg}}, j \in \Omega _{p o s}}} e^{\gamma \left( s_i-s_j+m\right) }\right) . \end{aligned}$$

In which *i* is a negative sample in set $\Omega _{neg}$, *j* is a positive sample in set $\Omega _{pos}$, $\gamma$ is a scaling factor, $s_i$ is a non-target score, $s_j$ is a target score and *m* is an interval.

3 $$\begin{aligned} \log \left( 1+\sum _{(i, j) \in N_\alpha } e^{s_\alpha (i, j)}\right) +\log \left( 1+\sum _{(i, j) \in P_\alpha } e^{-s_\alpha (i, j)}\right). \end{aligned}$$

The loss function is suitable for multi-label classification problem with large total number of categories and small number of target categories. It reduces the multi-label classification problem into the difference between target and non-target scores. In addition, because the number and types of entities in each sentence are not fixed, a threshold $s_0=0$ is set to determine which classes are exported, targets with score greater than 0 are exported.

In experiments, the specific loss function is the Eq. (3). Of these, all types of $\alpha$ entities head and tail collections (*i*, *j*) constitute positive sample sets $P_\alpha$, and all types of non-$\alpha$ entities or non-entity head and tail collections (*i*, *j*) constitute negative sample sets $N_\alpha$. Among them, $s_\alpha (i,j)$ represents the score of an $\alpha$ entity in the input sequence *S* consisting of a subset of elements *i* to *j*.

### Adversarial training

Adversarial training improves model robustness to malicious samples. Moreover, as a regularization method, it slows model overfitting and improves generalization^22^. As shown in Fig. 1, in the AT-CBGP model, the embedding vector encoded by Chinese BERT-base is $X = {x_1,x_2,...,x_n}$, and we add perturbed $\Delta x$ to the original embedded *X* to generate the vector $X_{adv}$, which is calculated as follows:

4 $$\begin{aligned}{} & {} \Delta x=\grave{o} \cdot \frac{g}{\Vert g\Vert _2}, \end{aligned}$$

5 $$\begin{aligned}{} & {} g=\nabla _{X} L(X, y; \theta ), \end{aligned}$$

6 $$\begin{aligned}{} & {} X_{adv} = X + \Delta x, \end{aligned}$$

in which, $\grave{o}$ is the superparameter representing the degree of disturbance, *g* is the gradient of the loss function, $L(X,y,\theta )$ is the loss function and $\theta$ is hyper parameter.

## Experiments

### Dataset and pre-processing

Four Chinese NER datasets were used to evaluate the model, including: (1) OntoNotes 5^34^, (2) MSRA^35^, (3) Resume^14^, (4) Weibo^36,37^. Among them, OntoNotes 5 and MSRA are datasets in the news domain, and Resume and Weibo are datasets in the social media domain.

The OntoNotes 5 dataset segmentation used in this article is consistent with the paper^35^. The MSRA dataset consists of only the training and test sets, in which the same number of samples as the test sets are taken as validation sets, divided into 8:1:1. Resume dataset segmentation is consistent with the paper^14^. On the Weibo dataset, this article uses the same dataset segmentation as the paper^37^. Based on the size of the corpus, this article classifies Onto Note 5 and MSRA as large datasets and Resume and Weibo as small datasets. Table 1 shows more details about the experiments for each dataset.

**Table 1:** Information of datasets.

Datasets	Training (k)	Validation (k)	Testing (k)	Char avg.	Char STD
OntoNote 5	20.4	3.1	2.3	44.1	28.4
MSRA	23.6	3.0	3.0	51.7	28.6
Weibo	0.73	0.14	0.16	61.5	38.9
Resume	3.7	4.3	4.6	32.7	25.2

### Experiments configuration

All of our experiments were performed on a single NVIDIA Tesla P100 graphics card with 16G storage. Table 2 shows the model parameters. During training, the model used Adam as an optimization function and set the learning rate at 5e−5, using Chinese BERT-base as Encoder, with 12 layers of Chinese BERT-base and 768 dimensions of hidden layers. For large datasets OntoNotes 5 and MSRANER, the batch size parameter is set to 32; For small datasets ResumeNER and WeiboNER, the batch size parameter is set to 64. Due to the different sentence lengths in different datasets, the maximum sentence length in this paper is 256. To highlight the improved performance of the models presented in this article, the following models are selected as Baseline. In addition to the following Baseline, we compared the results with other SOTA models.

1. *Lattice LSTM* Lattice LSTM uses word-level and character-level information.
2. *LR-CNN* LR-CNN uses CNN to encode sentences, and proposes the rethinking mechanism to resolve lexical conflicts, and uses CRF decoding.
3. *LGN* LGN uses global information from sentences to model and aggregate to solve the problem of lexical conflict.
4. *CGN* CGN uses the Graph Attention Network to combine dictionary and character information in the encoding layer.
5. *FLAT* FLAT uses the new relative position encoding to represent the positional relationship between words.
6. *LEBERT* LEBERT uses the BERT adapter to improve Chinese sequence tagging.

**Table 2:** Model parameters.

Parameter	Value
Transformer layer	12
Hidden layer dimension	768
Optimizer	Adam
Learning rate	5e−5
Training batch size	32/64
BERT dropout	0.5
Max sentence length	256
Max Epoch	50

### Analysis

In experiments, we use the rotary relative position encoding mechanism to solve the problem that the traditional attention mechanism lacks relative position information, and the global pointer network to solve the problem that the traditional conditional random field cannot capture global information, and add the adversarial training module. In this section, different model performance across four public datasets is analyzed and discussed. In this article, the best model on the validation set is used to evaluate the test set.

MSRA: Table 3 presents the results of experiments on the MSRA dataset. Here, LSTM based methods utilizing character-level and word-level feature information, and Lattice LSTM, LR-CNN, and LEBERT models utilizing word-level information. Compared with others, our model shows similar performance to the LEBERT model on MSRA without utilizing dictionary, which indicates the validity of this model.

Resume NER: Table 3 shows experimental results on the Resume dataset. Table 3 shows that LEBERT gets the highest F1 score, while our model represents a 0.42% improvement over LEBERT in F1. Compared with the model with dictionary, our model can achieve the same without extra support.

**Table 3:** Experiment results on MSRA & resume NER.

Model	MSRA			Resume NER		
	P (%)	R (%)	F	P (%)	R (%)	F
Lattice LSTM^14^	93.57	92.79	93.18	94.81	94.11	94.46
LR-CNN^15^	94.50	92.93	93.71	95.37	94.84	95.11
LGN^38^	94.19	92.73	93.46	95.28	95.46	95.37
Word LSTM	90.57	83.06	86.65	93.72	93.44	93.58
+Char+bichar	91.05	89.53	90.28	94.07	94.42	94.24
Char LSTM	90.97	86.96	88.81	93.66	93.31	93.48
+Bichar+softword	92.97	90.80	91.87	94.53	94.29	94.41
CGN^16^	94.01	92.93	93.47	–	–	94.12
LEBERT^39^	–	–	95.70	–	–	96.08
FLAT^19^	–	–	94.12	–	–	95.45
AT-CBGP(our)	95.33	95.16	95.12	96.38	96.63	96.48

Weibo NER: Table 4 shows experimental results on Weibo NER datasets. Among them, the overall F1 column represents the average value of the named entity F1 and the generic entity F1. LEBERT used the dictionary adapter to inject dictionary information between the transfer layers, achieving the highest F1 score. Compared with others, our model achieves an F1 score of 71.19% without external dictionary information.

Onto Notes 5: This article uses the Onto Notes 5.0 version dataset. Most of existed works, such as^38^ use, the Onto Notes 4.0 version dataset. Therefore, this article draws on the results of Ref.^35^ on the Onto Notes 5 version, and the comparison model will be slightly different from the previous three public datasets. Table 4 shows the experimental results on the Onto Note 5 dataset. Models such as Lattice LSTM and LR-CNN in the first four lines of Table 4 incorporate lexical information in various ways. TENER and FLAT improve model performance by improving relative location coding. FLAT reached 77.35% of the F1 score, and our model is 2.85% higher in F1 than FLAT.

**Table 4:** Experiment results on Weibo & OntoNote 5.

Model	Weibo			OntoNote 5		
	N (%)	G (%)	E (%)	P (%)	R (%)	F
Joint(cp)^36^	51.96	61.05	56.05	–	–	–
F-Score^40^	50.60	59.32	54.82	–	–	–
Adversarial SA^41^	54.43	57.53	58.70	–	–	–
JT-LSTM^42^	55.28	62.97	58.99	–	–	–
Unified Model^43^	54.50	62.17	58.32	–	–	–
CAN-NER	55.38	62.98	59.31	–	–	–
LEBERT^39^	–	–	70.75	–	–	–
TNEER	–	–	–	74.53	76.23	75.37
AKE^35^	–	–	–	76.22	78.29	77.24
CGN^16^	56.45	68.32	63.09	–	–	73.80
Lattice LSTM^14^	53.04	62.25	58.79	75.20	77.13	76.15
LR-CNN^15^	57.14	66.67	59.92	75.80	76.23	76.01
LGN^38^	55.34	64.98	60.21	75.58	76.75	76.16
FLAT^19^	–	–	60.32	76.26	78.46	77.35
AT-CBGP(our)	–	–	71.19	79.05	81.74	80.20

## Discussion

Table 5 show the F1 score of ablation experiments on four public datasets, w/o represents the removal of corresponding modules from the model. Statistics show that F1 score decreased by 1.98% and 7% on the Onto Note 5 and Weibo, respectively, using only models that removed Adv and ROPE for physical identification. In the Weibo dataset, in particular, the model that removed ROPE experienced a 3.98% decrease in F1 score on Weibo dataset, the largest decrease of any dataset. This due to the small size of the dataset, which can lead to over-fitting during training.

Table 5 shows the results of ablation experiments on the Resume dataset. Compared to the AT-CBGP model, the model with Adv removed showed a slight improvement in F1 score, but the model with Adv added showed a 96.93% improvement on indicator R, a better recall rate and a lower performance on indicator P. The following conclusions can be drawn from the analysis of Table 5: (1) Effect of improved attention mechanisms on the model, and F1 score of the model were increased across multiple datasets after using the attention mechanisms of rotary relative position coding. This shows that relative location coding injects relative location information into the model, can make the model better distinguish the distance information between entities and enhance the ability to distinguish the distance between entities. (2) With regard to the impact of adversarial training on the model, the model with adversarial training shows improved performance across datasets, which proves that adversarial samples can improve model robustness and improve the model’s ability to identify malicious samples.

**Table 5:** Ablation experiments results on OntoNote 5, MSRA, Weibo and Resume dataset.

Dataset		AT-CBGP	w/o Adv	w/o RoPE	w/o Adv & RoPE
OntoNote 5	P (%)	79.05	78.66	76.56	76.84
	R (%)	81.74	81.58	82.48	79.98
	F (%)	80.20	79.92	79.20	78.22
MSRA	P (%)	95.62	95.88	94.71	93.62
	R (%)	94.70	93.65	91.51	93.68
	F (%)	95.12	94.70	92.62	93.61
Weibo	P (%)	74.11	69.71	67.74	66.34
	R (%)	68.70	69.98	67.09	62.59
	F (%)	71.19	69.80	67.21	64.19
Resume	P (%)	96.03	96.38	95.46	95.68
	R (%)	96.93	96.63	97.12	96.64
	F (%)	96.48	96.50	96.28	96.15

## Conclusion

Entity recognition is an important subtask of knowledge map and natural language processing, and is of great significance to the construction of knowledge map. In this paper, a named entity recognition model AT-CBGP based on global pointer and adversarial training is proposed to improve the robustness and generalization of the model by using global pointer. Experimental results show that the model performs well on multiple open datasets. In future work, we will consider adding a wealth of lexical information to the model to improve its performance.

## Data Availability

The datasets used and analysed in the current study are available from the corresponding author on reasonable request.
